# Service delivery and the role of clinical pharmacists in UK primary care for older people, including people with dementia: a scoping review

**DOI:** 10.1186/s12875-024-02685-x

**Published:** 2025-01-14

**Authors:** Alice Burnand, Abi Woodward, Vladimir Kolodin, Jill Manthorpe, Yogini Jani, Mine Orlu, Cini Bhanu, Kritika Samsi, Victoria Vickerstaff, Jane Wilcock, Jane Ward, Greta Rait, Nathan Davies

**Affiliations:** 1 Research Department of Primary Care and Population Health, Centre for Ageing Population Studies, University College London, London, UK; 2 Centre for Psychiatry and Mental Health, Wolfson Institute of Population Health, QMUL, London, UK; 3 NIHR Policy Research Unit in Health & Social Care Workforce, King’s College London, London, UK; 4 NIHR Applied Research Collaborative (ARC) South London, King’s College London, London, UK; 5 Research Department of Practice and Policy, School of Pharmacy, University College London, London, UK; 6 Centre for Medicines Optimisation Research and Education, University College London Hospitals NHS Foundation, London, UK; 7 Research Department of Pharmaceutics, UCL School of Pharmacy, University College London, London, UK; 8 Research Department of Primary Care and Population Health, PRIMENT Clinical Trials Unit, University College London, London, UK; 9 Patient and Public Involvement, London, UK; 10 Centre for Evaluation and Methods, Wolfson Institute of Population Health QMUL, London, UK

**Keywords:** Pharmacy, Older people, Primary care, Medication, Care homes

## Abstract

**Objective:**

As populations age globally, there is increasing prevalence of multiple long-term conditions, such as dementia, leading to many challenges. The burden on health and care services, economic pressures, and the necessity for innovative policies to better support older people and people with dementia becomes paramount. This review explores how clinical pharmacists working in UK primary care support older people and people with dementia.

**Design:**

Scoping review.

**Method:**

This review was conducted following the framework for scoping reviews in accordance with the Joanna Briggs Institute (JBI) methodology. The search of Scopus, EMBASE, CINAHL, Web of Science, PsycINFO, and Cochrane was initially conducted in September 2022, and updated in August 2024. Searches included literature exploring the landscape of clinical pharmacy services for older people in the UK, focusing on roles and services delivered, perceptions, and experiences.

**Results:**

A total of 30 articles were included. These detail the multifaceted responsibilities of clinical pharmacists in primary care for older people. Stakeholder perspectives, including healthcare professionals and patients, emphasised the positive outcomes of clinical pharmacist involvement, from reducing other practitioners’ workloads to improving patient safety. However, communication gaps, concerns about competence from other healthcare professionals, and the need for clear role definitions emerged as challenges. Research focused on the experiences of underserved groups, such as people with dementia or from minority ethnic backgrounds, is lacking.

**Conclusions and implications:**

The review enhances our understanding of the primary care clinical pharmacist service in the UK and identifies gaps in evidence, emphasising the need for empirical studies on the experiences of older people with cognitive impairment and those from minority ethnic backgrounds. It provides insights into what makes an effective clinical pharmacist service, such as training and communication, which may help to inform international policy and practice and improve service provision globally.

**Supplementary Information:**

The online version contains supplementary material available at 10.1186/s12875-024-02685-x.

## Introduction

Globally the population is ageing, and more people are living with multiple long-term conditions (LTCs) [1]. This demographic shift and rise in LTCs, such as dementia, brings many challenges for a country’s societal, economic, and health and care systems. There remains increased demand for health and care services, economic pressures, and the need for innovative policies to better support the well-being of older people [2]. In the UK, LTCs account for 70% of the National Health Service (NHS) healthcare budget [3] and the number of patients being diagnosed with multiple LTCs, is increasing, as highlighted in the UK Chief Medical Officer’s 2023 Annual Report [4]. The population of people over 65 in the UK is projected to make up nearly 25% of the population by 2043, up from 19% in 2019 [5]. This is also causing an upward trajectory in the prevalence of dementia, as it typically affects older people. The economic impact of dementia is vast, is projected to be the costliest health condition by 2030 and is expected to cost £47 billion by 2050 [6, 7].

Primary care services form the bedrock of the UK’s NHS however they are facing growing challenges to support the needs of patients by providing care and treatment and remain an effective gatekeeper system. Primary care teams in the UK generally consist of various healthcare professionals including specialist nurses, physiotherapists, social prescribers, and pharmacists, who work collaboratively to ensure that patients receive person-centred, holistic, and coordinated healthcare. Team compositions vary dependent on the needs of the patient population and the specific services offered.

Pharmacists have an integral role in many primary care teams, and in recent years their work has increasingly become more clinically oriented, such as duties in prescribing and deprescribing medication [8]. Deprescribing includes the planned and supervised dose reduction, or complete stop, of medication that may cause harm or no longer be of benefit [9]. The introduction in England of clinical pharmacists working in general practices began as a pilot study in 2015, initiated by NHS England [10]. Independent evaluations concluded the addition of clinical pharmacists made a substantial contribution to the primary care skill mix, as well as enhanced patient safety and increased healthier lifestyles [11]. It was argued that they should form an “integral part of general practice” [12]. In 2019 NHS England agreed a five-year general practitioner (GP) framework providing funding to support the recruitment of clinical pharmacists in primary care networks [13], as well as further investment with an additional 1,500 clinical pharmacists in 2020/21 [14] and 7,500 clinical pharmacists in 2024 [13, 15].

Numerous studies have highlighted the association between polypharmacy, defined as the regular use of five or more medications at one time [16], and inappropriate medication use among older people [17–19]. This may lead to various detrimental outcomes such as reduced quality of life, adverse reactions to medications, unexpected healthcare utilisation and earlier care home admission, morbidity, and mortality [20]. Overprescribing, where patients are prescribed medications that are inappropriate and harm outweighs benefits, may disproportionately affect Black, Asian and minority ethnic communities in the UK, and polypharmacy increases with relative deprivation [21]. Excessive polypharmacy is also common in care home residents [22] who are often older, frail people with complex care needs due to multiple LTCs and cognitive impairment [23]. In the UK there are two types of care homes, residential which have no onsite physician or nursing staff, but are supported by external health care services or nursing homes with onsite nursing staff. Clinical pharmacists in primary care play a large role in supporting care homes. By actively addressing polypharmacy and optimising medication management primary care, clinical pharmacists have valuable potential for improving the wellbeing of older people, those living with dementia, and those from minority ethnic backgrounds.

Older people and people with dementia often experience complex medication regimes and polypharmacy, increasing their risk of adverse drug events. Clinical pharmacists have expertise in medication management, but their role in supporting these populations in the community is underexplored. This scoping review sought to identify existing evidence and identify knowledge gaps in this field. This review has the potential to enhance our understanding of the role of clinical pharmacists in primary care, what are the needs of these groups in clinical practice, and how research can support this. In turn this can improve outcomes for older people and people with dementia.

### Primary review question

What research has been conducted on clinical pharmacy services used in primary care for older people (over the age of 65 years) in the UK?

### Secondary review questions

- What is the role of clinical pharmacists, and what models of clinical pharmacy are used in primary care for older people in the UK?
- What is the role of clinical pharmacists, and what models of clinical pharmacy are used in primary care for people living with dementia in the UK?
- What are the perceptions of health and professionals of the role (including benefits, challenges and purpose) of clinical pharmacists who are involved in the care of older people in primary care in the UK?
- What are the experiences of older people and their carers (family and friends) who have encountered clinical pharmacists in primary care in the UK?
- What are the experiences of older people from minority ethnic groups and those from backgrounds of social deprivation in the UK?

## Method

### Design

This review was conducted following the framework for scoping reviews in accordance with the Joanna Briggs Institute (JBI) methodology [24] and reported according to the Preferred Reporting Items for Systematic Reviews and Meta-Analysis extension for scoping reviews (PRSMA-ScR) [25]. A study protocol has been published [26].

### Data sources

Preliminary searches of PROSPERO and Medline were conducted on the 04/08/2022 and no current or ongoing reviews were identified. A full search strategy was developed from existing literature and discussions with an information specialist from the University library for Medline, CINAHL Scopus, EMBASE, Web of Science, PsycINFO, and Cochrane (Appendix 1). Categories included primary care, clinical pharmacist, older people, and medication management with search terms such as primary care, primary healthcare, general practice and pharmacists (Appendix 2). This scoping review considered all primary research studies (qualitative and quantitative), systematic reviews, meta-analyses, letters to editors, commentaries, blogs and grey literature. The latter includes theses, dissertations, trade publications, national policy and guidelines, reports, websites, conference abstracts and posters, preprints, and others. We excluded any articles before 2015, as we acknowledge that some parts of the UK may have other service delivery models prior to this date. We did not include systematic reviews in this scoping review but checked references for all reviews that were relevant to the inclusion criteria.

The electronic databases were searched from 1 January 2015 to 30 August 2022and updated in August 2024 for peer-reviewed literature based in the UK only, as our review has a focus on the delivery, provision, and model specificity of clinical pharmacist services in the UK context. Grey literature was searched using Google in September 2023, and updated in August 2024, and using the key word searches identified for the electronic databases, we searched through all 9 pages for eligible articles to be included. Articles were also identified via hand searching the recent lists of included articles, and authors were contacted for further information and missing data. Citation tracking was conducted through Google Scholar.

Searches vary slightly from the published protocol [26] as we made small refinements as our thinking developed and we became more familiar with the literature, as recommended to be good practice for scoping reviews in the JBI guidance [24].

### Data screening

**Fig. 1:**
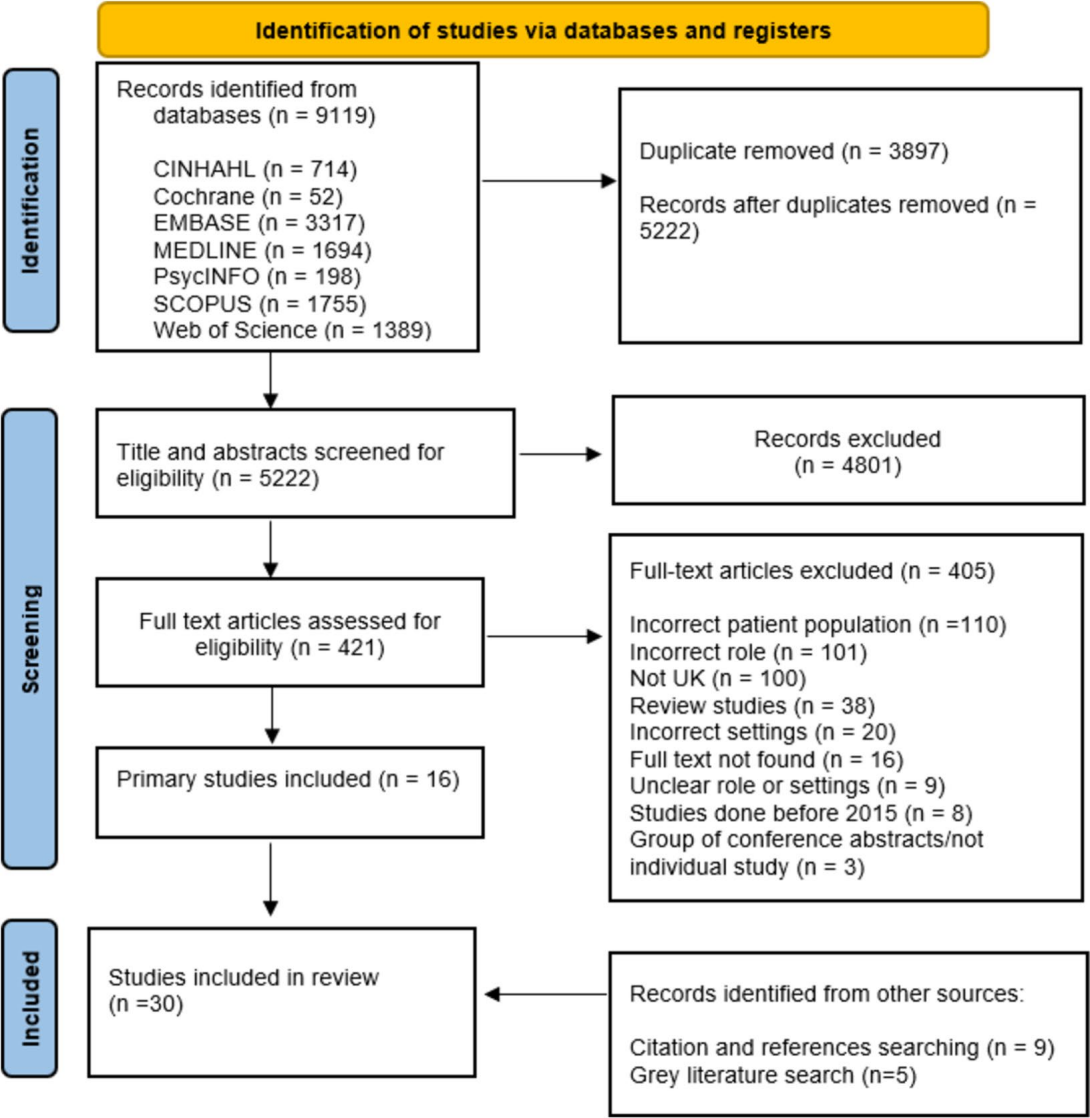
PRISMA study selection flowchart

 All identified references were collated and uploaded into Endnote, duplicates were removed, and the remaining articles imported into Rayyn for screening. One researcher (VK) screened all the title and abstracts utilising the inclusion criteria (Table 1) and a second researcher (AB) screened a random 10%. Any discrepancies were discussed (VK and AB), with a third researcher involved if needed (ND). The full texts were retrieved and screened independently by two researchers (VK, AB or AW). Discrepancies were discussed and the eligibility criteria was revised as needed. Authors of papers were contacted to request missing or additional data, as for example with some papers, the role of the pharmacist or the setting was unclear. If a response was not received, they were excluded. The reasons for exclusion during the full-text screening are reported in Fig. 1.

**Table 1:** Inclusion/exclusion criteria for screening

	Included	Excluded
Population	Older people (65 years and above) or if paper describes them as older people.	Mixed population with no separate analysis on older people.
	Any health and care professionals caring for older people.	Those under 65 years.
	Family carers of older people.	
Concept	Clinical pharmacist role.	Community pharmacy.
	Clinical pharmacy in primary care.	Unclear role, when a definition is not provided, or when a paper refers to a pharmacist, non-medical health professional, supplementary prescriber but role and pharmacy type are unclear.
	Focus on what clinical pharmacy services are delivered and how.	
	Experiences of services from any professionals, family carers and older people’s perspectives	
Context	Primary care.	Studies outside the UK.
	The UK.	Studies prior to 2015.
	Published since 2015 and the introduction of the clinical pharmacist in general practice.	Studies of the community pharmacy role.
	Care homes.	Dentistry and optometry services.
		Non-English language.
Design method	Any primary research study.	All reviews that met the inclusion criteria were excluded but had references checked.
	Grey literature.

### Data extraction

The data from the included sources were extracted by two independent reviewers (VK and AB) using a data extraction tool developed by the research team using Microsoft Excel. On completion, any differences in the data extracted by VK and AB were highlighted, discussed, and reviewed again. The data from the extraction tool was then narratively summarised and tabulated according to the study it was identified from and the research methods.

### Synthesis and critical appraisal

Aligning with the guidance of scoping reviews, quality assessments were not conducted [27]. Results were synthesised using a descriptive narrative synthesis structured using the research objectives and using the principles of codebook thematic analysis [28]. Papers were read and re-read several times to familiarise ourselves with the content of the paper. Two papers were initially coded by AB, applying codes to the results sections of the papers. Codes were developed deductively informed by the research objectives. These initial codes were discussed and refined among three authors (AB, ND, AW). Codes were then applied to the remaining papers results sections by AB. All codes were discussed, and overarching themes were developed to group the codes and present the main findings in relation to the objectives. Theme title and descriptions were discussed regularly by three members of the team (AB, ND and AW) and agreed upon by all authors discussed in two meetings.

## Results

### Study inclusion

The initial search strategy identified 8405 records from the database search, after de-duplication, title, abstract and the full text screens, 30 articles were included (Fig. 1).

### Characteristics of included studies

The general characteristics of the included articles are provided in Table 2. The papers included quantitative (*n* = 10), qualitative (*n* = 8), mixed (*n* = 7) designs. A small number were identified from the grey literature search, which include a blog post and website articles (*n* = 5).

**Table 2:** General characteristics of included studies

**Quantitative**							
**Author**,** year**,** location**	**Participant demographics / population**	**Primary care setting**	**Condition(s) or medicine(s) or other area(s) of practice**	**Design methods (study design/ source type)**	**Aim/objectives**	**Main results**	**Models of care**
**Alharthi**,** M. et al.**,** (2022). United Kingdom** [29]	PIPS *n* = 22	Care homes	Deprescribing	Peer-reviewed publication. Cluster RCT.	To test the hypothesis that contextual factors influenced the likelihood of deprescribing by PIPs. Part of CHIPPS.	Deprescribing The number of residents and PIP’s employment within a medical practice were positive predictors of deprescribing. Experience was not related to deprescribing. From the 566 medicines management interventions undertaken by PIPs, 50.2% were deprescribing of which 97.2% remained deprescribed at 6 months.	Enhancing existing models of care - PIP medication management within care home.
**Baqir et al.**,** (2017). United Kingdom** [30]	Residents *n* = 422 Age: 85.5 years Female: 77.7%	Care homes	Medication reviews and deprescribing	Peer-reviewed publication. Retrospective analysis of the 2012 Shine Medication Optimisation Project [31].	To investigate whether medicines review led by pharmacists would lead to deprescribing, and to assess the reasons for and impact of deprescribing.	Deprescribing The mean number of medicines stopped was 2.36 (SD 1.53). There was no statistical difference between amount of medicine stopped by pharmacists (53.4% stopped) and where GPs were involved (51.9%) (*p* = 0.9702). 56.8% of medicines were stopped as they had no current indications, 15.9% stopped by patient choice, 8.7% stopped as no longer appropriate and 6.5% for safety reasons.	Enhancing existing models of care – PIP medication management within care home.
**Holland**,** R. et al.**,** (2023). United Kingdom** [32]	Patients *n* = 882 taking at least one prescribed drug. Age: 85 years Female: 72%	Care home	Falls risk and deprescribing	Peer-reviewed publication. Cluster RCT.	To estimate the effectiveness, and safety of pharmacy independent prescribers in care homes. Part of CHIPPS.	Deprescribing and harm prevention The rate of falls between the intervention group and control group was not significant (95% CI 0.66 to 1.26). Hospital admissions and Barthel score were not significantly different between groups, but the Drug Burden Index was significant, which suggests effective deprescribing took place. No adverse events or safety concerns were identified.	Enhancing existing models of care - intensive weekly PIP visits to care home.
**Hunt**,** V. et al.**,** (2018). United Kingdom** [33]	Patients *n* = 173 Age: 70 years Females: 66.9% Ethnicity: white British *n* = 173 (100%).	General practice	COPD	Peer-reviewed publication. A non-randomised controlled pilot study.	To evaluate the impact of pharmacist intervention on exacerbations and respiratory hospitalisations.	Harm prevention and condition management Many patients accepted pharmacist input. 72.1% of the intervention group received dose changes; 52.3% had medicines stopped/started and 24.4% received an expedited review at the specialist respiratory consultant clinic; 53.5% were referred to other healthcare services. At follow-up, there was a significant difference in exacerbations with 63.5% of intervention group and 86.2% in control group (*p* = 0.001). There were also fewer respiratory hospitalisations in intervention (45.3%) vs. control participants (76.7%) *p* < 0.001. The intervention may have been cost saving, based on costs of staff time, clinic attendance, and hospitalisations.	New model of care - clinical pharmacist with an interest in respiratory therapeutics, working 3 days per week, collaborating with a specialist respiratory physician, for over 1 year.
**Hurley et al.**,** (2024). United Kingdom** [34]	Residents *n* = 99 Age: 86.24 years (SD 6.9) Female: 76.7%	Care home	Deprescribing	Prospective, unblinded, non-randomised, intervention study.	To demonstrate the impact that pharmacist-led application of STOPPFrail (Screening Tool of Older Persons’ Prescriptions in Frail adults with a limited life expectancy) could have on reducing potentially inappropriate medications (PIMs) and clinical outcomes for frail older adults in nursing homes.	Deprescribing The intervention significantly reduced the number of regularly prescribed medications, with 1 in 10 medications discontinued post review, which remained significant at 6 months. The most frequent PIMs were medications without a clearly documented indication (29.6%) and vitamin D (16.9%). 203/348 recommendations provided to GPs were accepted and 193 (55%) implemented. The decrease in number of medications resulted in a 7.5% reduction in monthly medications costs, although not maintained at 6 months. No significant differences were found for health-related outcomes, including unplanned hospital admissions, emergency department visits, falls, and QoL. No deaths were related to deprescribed medications, confirming the safety of these pharmacist-led interventions.	Enhancing existing models of care – pharmacist medication management and deprescribing within care home.
**Oboh**,** L. et al.**,** (2018). United Kingdom** [35]	Patients *n* = 143 Age: 78 years Female *n* = 87 (61%).	At patient’s homes	Frail and complex patients with multiple LTCs. The mean number of conditions per patient was 9.	Peer-reviewed publication. Retrospective description of a new service.	To describe the new integrated care clinical pharmacy service and to provide information regarding the medicines-related support provided.	Condition management A total of 376 medicines-related problems were identified, due to: 7% supply issues, 29% compliance issues and 64% clinical issues. The service supported current health policy priorities by identifying and addressing a range of medicines related problems.	New model of care - an integrated care clinical pharmacy service as part of the health and social care team, which visits frail, older people in their own homes.
**O’Mahony et al.**,** (2024). United Kingdom** [36]	Patients *n* = 1471 Age: 76.0 years (SD ± 9.7)	General practice	Hyperpolypharmacy (≥ 10 medications)	Peer-reviewed publication. Economic evaluation cost-benefit analysis	To conduct a cost-benefit analysis of a pharmacist-led medicines review service across multiple Irish general practice settings involving patients with hyperpolypharmacy and/or at high risk of medicines-related harm.	Medication optimisation By conducting person-centred medicines reviews, general practice pharmacists can significantly reduce costs for patients at high risk of medication-related harm. Based on 1471 patients (88.4% with hyperpolypharmacy), the cost of service delivery was €153 per review. Net cost savings ranged from €198 to €288 per patient review and from €73,317 to €177,696 per annum per pharmacist. Moreover, there were net cost savings of €651–€741 per review, and annual savings of €240,870–€457,197 per annum per pharmacist.	Enhancing existing models of care – pharmacist medication management and deprescribing within general practice.
**Ritchie et al.**,** (2024). United Kingdom** [37]	Residents *n* = 21 Age 85.0 years (6.5) Female: 63.6%	Care home	AF	Peer-reviewed publication. Individually randomised, prospective pilot and feasibility study.	To assess the feasibility of pharmacist-led medicines optimisation in care home residents, based on the ABC (Atrial Fibrillation Better Care (ABC: Avoid stroke; Better symptom management; Cardiovascular and other comorbidity management)) pathway compared to usual care.	Medication optimisation It was feasible to use the ABC pathway as a framework for pharmacist medication review, but a ceiling-effect was observed whereby most residents’ medications were already optimised as much as possible according to the ABC pathway. Implementation rates of pharmacist recommendations by GPs were around 48%. Overall ABC adherence rates did not change after pharmacist medication review; 3/11 residents were adherent to all three components of the ABC pathway, 9/11 resident’s adherent to the ‘A’ component and the ‘B’ component, compared to 3/11 residents for the ‘C’ component. However, the study was unable to draw any conclusions on the effect of the intervention on health-related outcomes because overall ABC adherence did not change and the study was underpowered.	Newmodel of care – pharmacist-led intervention utilising the ABC pathway for people with AF.
**Savickas**,** V. et al.**,** (2020a). United Kingdom** [38]	Patients *n* = 604 Age: Median age 73 years Female: 57.3% Ethnicity: White British 96.9%. Also, White Irish, White American, White Dutch, White Other and Other.	GP practice	AF	Peer-reviewed publication (research). Cross-sectional feasibility study.	To determine the feasibility of clinical pharmacists to screen for AF using digital technology (single-lead ECG (_SL_ECG)) and pulse palpation.	Diagnosis The sensitivity and specificity of clinical pharmacists diagnosing AF using pulse palpation was 76.9% and 92.2%, respectively. This rose to 88.5% and 97.2% with an _SL_ECG and helped identify new non-AF cardiovascular diagnoses. At follow up, 4 participants (0.7%) were diagnosed with new AF and three (0.5%) were started on anticoagulation. Participant feedback showed, participants were highly satisfied with the consultation and thought AF screening was important.	New model of care - single-time point screening strategy for clinical pharmacists to screen and detect AF.
**Virdee**,** M. et al.**,** (2017). United Kingdom** [39]	Patients *n* = 497 Age: 75.5 years Female: 41.4% Ethnicity: Unknown	GP practice	AF	Peer-reviewed publication. Clinical audit of practice.	To quantify the level of anticoagulation utilisation in patients according to evidence-based guidelines and to assess the impact of a pharmacist-led intervention to optimise therapy.	Medication optimisation 77% of pharmacist recommendations to a GP were agreed without modification. 41% patients were candidates for anticoagulation, 51% were not eligible, 21% were anticoagulated. Intervention corrected overuse of anticoagulants to evidence-based guidelines for stroke prophylaxis. There remains a need to consider patients whose anticoagulants are contraindicated and those refusing anticoagulants.	Enhancing existing models of car – use of GRASP-AF tool in audits according to NICE guidelines.
**Qualitative**							
**Author**,** year**,** location**	**Participant demographics / population**	**Primary care setting**	**Condition(s) or medicine(s) or other area(s) of practice**	**Design methods (study design/ source type)**	**Aim/objectives**	**Main results**	**Models of care**
**Birt et al. (2023). United Kingdom** [40]	PIPS *n* = 14 GPs *n* = 9 Care home managers *n* = 9 Care home staff *n* = 6	Care homes	Medication management and deprescribing	Peer-reviewed publication. Secondary analysis of interviews.	To understand the application of the PIP role and the perceptions of PIPs, GPs, and CH staff regarding the effects of PIP activities on CH medical processes and resident well-being. Part of CHIPPS	Deprescribing The results provide more understanding of the PIP role within the wider primary care team, and most PIPs and GPs stated PIPs should have a continued role within care homes to assist with the management of medicines. Participants reported benefits from PIP involvement to do with increased safety, wellbeing and streamlined care home procedures. PIPs were also seen with specialised clinical knowledge. Trust between all stakeholders improved outcomes. However, a few participants were still unclear about their specific responsibilities and PIPs role need to cover more than reviewing medications to maximise impact.	Enhancing existing models of care - PIP medication management within care home.
**Birt**,** L. et al.**,** (2022b). United Kingdom** [41]	Primary care pharmacists *n* = 16 GPs *n* = 6 Care home staff *n* = 7	Care homes	Deprescribing	Peer-reviewed publication. Semi-structured interviews.	To explore beliefs and practices of deprescribing in care homes that act as enablers and barriers. Part of CHIPPS.	Deprescribing Two themes were identified: ‘Structures and systems affecting deprescribing including’, which describes the context in which deprescribing happened and the team involvement and ‘Balancing risks when deprescribing’, which is the perception of risks and social barriers were reduced by understanding the medical background of each of the residents. Authors reported clinical pharmacists can lead the process of deprescribing, by having competence and willingness and stakeholders were in favour of deprescribing to reduce problematic polypharmacy.	Enhancing existing models of care - PIP medication management within care home.
**Cunningham**,** Y. et al.**,** (2021). United Kingdom** [42]	Health care professionals *n* = 25 Patients *n* = 22 Ethnicity: most of sample was Caucasian.	General practice	Anticholinergic burden	Peer-reviewed publication. Interviews and focus groups	To explore the views of key stakeholders (patients, the public, and HCPs) regarding the acceptability, design, and conduct of an anticholinergic burden (ACB) reduction trial.	Medication optimisation There was consensus among the different groups that key points to consider with such a trial included: (1) ensuring patient engagement throughout to enable concerns/potential pitfalls to be addressed; (2) ensuring clear communication to minimise potential misconceptions about the reasons for ACB) reduction; and (3) contacts for patients to access throughout the trial to address concerns.	New model of care – designing a new anticholinergic burden reduction trial.
**Fowler et al.**,** (2021). United Kingdom** [43]	Participants *n* = 9, included pharmacists, pharmacy technicians, care home managers and one manager (from CCG).	Care homes	Medication management	Peer-reviewed publication. Semi-structured interviews.	To investigate the outcomes of the MOCH team in medicines management.	Medication management Authors highlighted the importance of transparency alongside person-centred care to make explicit the reason for taking a medication, and discussions with a range of stakeholders about the continuing need for medications. The outcome of the evaluation includes insights into a new area of pharmacy practice in community, based on the skills, knowledge, and experience of pharmacists and pharmacy technicians working in the care home sector.	Enhancing existing models of care - MOCH intervention (clinical pharmacists and pharmacy technicians working in care homes).
**Lane et al.**,** (2020). United Kingdom** [44]	Focus groups *n* = 72 participants Stakeholder focus groups (total *n* = 85) Interviews were held with GPs, pharmacists, pharmacy technician, care-home managers, care-home staff, residents, and relatives.	Care homes	Medication reviews	Peer-reviewed publication. Focus groups and semi-structured interviews.	To explore stakeholders’ views on the issues and barriers that PIPs might address, to inform a service specification for PIP intervention. Part of CHIPPS.	Medication management All participants welcomed introducing a PIP into care homes in principle but conditional on a clearly defined PIP role communicated to stakeholders; collaboration; trust and effective communication. To embed a PIP service everyone must “understand each other’s systems”.	Enhancing existing models of care - PIP medication management within care home.
**McCahon et al.**,** (2022). United Kingdom** [45]	10 general practices. Patients *n* = 21 Female *n* = 10 Age 73 years (range 59–88).	General practice	Medication management and polypharmacy	Peer-reviewed publication. In-depth interviews.	To gain a better understanding of patient perceptions and experiences of medication review as undertaken in routine general practice.	Communication Whilst pharmacists were thought to have greater knowledge of medicines than GPs, several participants were unfamiliar with the role of pharmacists within general practice and expressed a lack of confidence in the clinical skills and knowledge of pharmacy professionals. Opinions and perceptions were primarily shaped by patient experiences with community pharmacists rather than direct interactions with pharmacists working in primary care settings.	Enhancing existing models of care – pharmacist-led medication reviews in general practice.
**Savickas et al.**,** (2020b). United Kingdom** [46]	Patients *n* = 25 Age: 68–73 years Female: 48% Ethnicity: White British (92%). Clinical Pharmacists (*n* = 4) Female: 50% GPs (*n* = 9) Female: 100%	General practice	AF	Peer-reviewed publication. 3 stakeholder focus groups.	To explore the perspectives of stakeholders involved in the ‘Pharmacists Detecting Atrial Fibrillation’ (PDAF) study to understand facilitators and barriers to pharmacist-led AF screening in general practice.	Diagnosis Three main themes were identified:1) knowledge and awareness; 2) prioritisation of resources; and 3) environmental considerations. The public’s lack of awareness of AF-related risks and pharmacist-led screening services was highlighted. Pharmacists were perceived as an underutilised educational resource and enabled access to screening to reducing GPs’ workload. Participants agreed that AF screening should be involved in health checks and vulnerable groups should be prioritised, such as care home residents. Patients favoured the GP over the community pharmacy where concerns were raised.	New model of care – pharmacists opportunistic screening for AF during influenza vaccinations.
**Wright et al.**,** (2021). United Kingdom** [47]	Phase 1: 72 patients in interviews and focus groups. Phase 2: 23 HCPs in focus groups and 4 in interviews. Phase 3: 53 attendees on the panel. Phase 4: 4 PIPs, GPs, and care homes, each with 10 consented residents were recruited.	Care homes	Pharmaceutical care safety	Peer-reviewed publication. Focus groups, expert panel consensus and feasibility testing.	To develop and feasibility test a training programme to enable PIPs to provide pharmaceutical care safely and effectively within the care home. Part of CHIPPS.	Training and medication optimisation A key finding the need for the PIP to develop relationships and understand the local context with the care home staff, medical practice, and community pharmacist. Authors reported differences in baseline knowledge of PIPs required inclusion of a Personal Development Framework and the provision of a mentor. Face-to-face training was said to focus on managing medicines for a complex older person, minimising prescribing costs and supporting people without capacity.	New model of care - using a Personal Development Framework, mentor, and assessor, supported with an underpinning knowledge pack and specific face-to-face training.
**Mixed**							
**Author**,** year**,** location**	**Participant demographics / population**	**Primary care setting**	**Condition(s) or medicine(s) or other area(s) of practice**	**Design methods (study design/ source type)**	**Aim/objectives**	**Main results**	**Models of care**
**Alves et al.**,** (2019). United Kingdom** [48]	Pharmacists (*n* = 25)	Care homes	Deprescribing	Peer-reviewed publication. Evaluation of interventions, rational for stopping medicines and impact of deprescribing.	To evaluate a novel pharmacy-led model of deprescribing unnecessary medications for care home patients.	Deprescribing and medication optimisation The 25 pharmacists undertook over 10,000 patient reviews (averaging 2 interventions per patient). Deprescribing accounted for over 50% of the total estimated financial drug savings, equating to nearly half a million pounds. Around one-sixth of all interventions were related to safety.	New model of care - Clinical pharmacists to offer at least one medication optimisation visit to as many care homes as possible across Somerset.
**Baqir et al.**,** (2015). United Kingdom** [49]	Residents (*n* = 422) Interviews with pharmacists, doctors and care home nurses, care home resident’s families.	Care homes	Medication reviews and deprescribing	Peer-reviewed publication. Non-randomised study and data analysis with interviews of stakeholders.	To develop a pragmatic approach to optimising medicines in care homes while involving residents in the decision making.	Medication reviews and deprescribing Over 12 months 422 residents were reviewed, and 1346 interventions were made in 91% of residents. The most common intervention (52.3%) was to stop medication. The net annualised savings were £184 per person reviewed. The project demonstrated that MDT reviews can safely reduce inappropriate medication in care home residents and GPs and care home staff valued contribution of the pharmacist.	Enhancing existing models of care – medication review involving MDT discussion and resident (and/or family).
**Birt et al.**,** (2021). United Kingdom** [31]	Interviews were completed with 14 PIPs, 8 GPs, 9 care home managers, 6 care home staff, and 1 resident. PIPs: Female: 82%	Care homes	Medication management	Peer-reviewed publication. Process evaluation to describe the intervention implementation, mechanisms of impact, outcomes, and contextual factors.	Process evaluation for the CHIPPS. To understand the exact way the intervention was implemented in practice, and the implications of this for the trial outcomes.	Medication management PIPs conducted medication reviews on residents, many involving deprescribing; 93.8% of changes were sustained at 6 months. 50.2% residents had a medicine was stopped linked to falls risk.; 16.8% to reduce a dose, 8.7% to change a medicine, 10.6% to start a medicine, 5% to increase a dose and 9.2% to initiate monitoring. Authors stated that all stakeholders reported some benefits from PIPs having responsibility for medicine management and identified no safety concerns. PIPs also reported an increase in their knowledge.	Enhancing existing models of care - PIP medication management within care home.
**Birt et al.**,** (2022a). United Kingdom** [50]	PIPs (*n* = 25) Post-training evaluation forms (*n* = 25) Post-intervention questionnaires (*n* = 16) Post-intervention interviews (*n* = 14).	Care home	Pharmaceutical care	Peer-reviewed publication RCT, evaluation forms, questionnaires, and interviews completed by GPs and care homes staff.	To evaluate a training programme designed to prepare PIPs to assume responsibility safely and effectively for pharmaceutical care of older people in care homes, within a randomised controlled trial. Part of CHIPPS.	Training and medication optimisation GPs and care home staff reported an increase in medication safety due to the PIP activity. Deprescribing could improve resident quality of life. Some PIPs identified new areas of learning and confidence. PIPs reported the training days and mentorship enabled them to develop a portfolio of competence for discussion during a viva with a GP. PIPs applied their new learning leading to perceived improvements in residents’ quality of life and medicines management.	New model of care – using Kaufman’s Model in training clinical pharmacists.
**Inch et al.**,** (2019). United Kingdom** [51]	GP practices (*n* = 4) Care homes (*n* = 6) PIPs (*n* = 4) Care home residents (*n* = 40) Age: 84 years Female: 60%	Care homes and GP practice	Medication management	Peer-reviewed publication. A single-arm, non-randomised open feasibility study of a PIP intervention. Data collection, questionnaires and focus groups/interviews.	To test and refine the service specification and proposed study processes to inform the design and outcome measures of a definitive randomised controlled trial (Holland et al., 2023). Part of CHIPPS.	Medication optimisation, training & support, and communication All participants expressed positive views about their experience of the new service. The main themes were improved patient care, improved patient safety and saving staff time and effort. PIP service was generally well received by the majority of stakeholders. The PIPs proposed a few minor changes to the service specification. They suggested ‘clarification of directions’ for medicines was ‘essential’ and that consultations should be conducted face-to face ‘where possible’.	New model of care – 4 h per week for 90 days per PIP per 10 residents for medication reviews, training, and communication.
**Martin and Murphy**,** (2017). United Kingdom** [52]	Patients and GPs	Living at home or in care home	Medication reviews	Published conference abstract. Data analysis and qualitative feedback from patients and GPs.	To provide a personalised medication review for older people within their own homes, including care homes.	Medication optimisation and harm prevention A total of 399 patients received a comprehensive medication review over a 10-month period. The pharmacists made 1738 recommendations and of these 70% were presented to the GP for consideration. Hospital avoidance interventions and direct cost-savings amounted to a calculated saving of £53,789.92 over the 10-month period. The service was well received by the patients and the multidisciplinary team.	Enhancing existing models of care – integrated community ageing team.
**Snell et al.**,** (2017). United Kingdom** [53]	34 GP practice. Patients at the practice who were prescribed more than 15 medicines. Age: Over 75 years Female: 56%	General practice	Polypharmacy	Peer-reviewed publication. Patient feedback questionnaire to capture both quantitative and qualitative data.	To investigate patient views about a patient-centred clinical pharmacist-led polypharmacy medication review service completed within GP practices.	Medication optimisation The main themes to come out of the qualitative responses were: (1) Process; the pharmacist’s personal approach, being listened to, the pharmacist’s advice and explanations, and questions or concerns being answered. (2) Outcome; increased confidence or knowledge about medication, general satisfaction with the service. Patients reported to appreciate pharmacists’ personal approach, advice, and explanation. 94% who had a medication-related concern beforehand were addressed, and 80% understood their medicines better after the review. A small number of negative comments stated the service was not useful for non-English speaking patients and those with impaired cognition.	Enhancing models of care – person centred care medication reviews in general practice.
**Grey Literature**							
**Author**,** year**,** location**	**Participant demographics / population**	**Primary care setting**	**Condition(s) or medicine(s) or other area(s) of practice**	**Design methods (study design/ source type)**	**Aim/objectives**	**Main results**	**Models of care**
**Barhey**,** M. (2017). United Kingdom** [54]	Patients over 75, taking 10 + medications	Care home	Older people with multiple medications and those with type 2 diabetes	Blog post	Clinical pharmacist in a care home for 3 months, to manage medication, improve medication adherence, identify medication errors, avoid adverse events and hospital admissions.	Medication optimisation and harm prevention Initial findings indicate a significant decrease in GP appointments within six months of the pilot’s implementation. There was expected reduced and simplified medication regimens, fewer falls, and lower hospital admission rates. They estimate annual savings of over £3,000 per patient.	Enhancing models of care – clinical pharmacists assigned to care homes to review resident’s care.
**Cudby and Syan**,** (2019). United Kingdom** [55]	N/A	Primary care	Long term conditions and polypharmacy	Website article	To explain the role of the Practice Pharmacist in supporting the management of frail older people in primary care.	Condition management and medication optimisation Authors state that the clinical pharmacist role is mainly in the management of patients with chronic diseases and medication optimisation reviews in polypharmacy, as required by the Network Contract Directed Enhanced Service (DES) specification.	Enhancing models of care – addressing polypharmacy with reviews and suggests framework of ‘Soar Beyond 3S’ about how pharmacists can improve frailty services.
**Khangura and Alani**,** (2016). United Kingdom** [56]	Post-discharge patients (*n* = 467) Age: over 75 years	Primary care	Polypharmacy	Website article	To address the issue of older patients being more susceptible to harm from polypharmacy and transfer between care settings can result in medication discrepancies.	Medication optimisation and harm prevention After eight months, 467 patients had been reviewed, with 521 interventions made. These include adverse drug reaction, compliance issues, queries with preadmission medicines and drug formulation issues. All patients in this study received a clinical pharmacy review in addition to the GP review, yet medication issues were still identified.	Enhancing models of care – remote or f2f appointment for medication review with clinical pharmacist.
**Wickware**,** (2018). United Kingdom** [57]	“Older adults”	Care homes	Reviewing residents care	Website article	To announce that pharmacists will be assigned to care homes in the “national roll out of a successful pilot”.	Medication optimisation and communication Author states that pharmacists involved were expected to participate in weekly rounds “for reviewing and planning a resident’s care”, alongside “the resident’s GP, the care home team and other members of the local multidisciplinary teams, such as nurse specialists”. The pharmacists will also offer emergency care out of hours.	Enhancing models of care – clinical pharmacists assigned to care homes to review resident’s care.
**Moroney et al. (2018). United Kingdom** [58]	“Older adults”	Patients at their residences or at the GP surgery	Polypharmacy and medication review	Website article	To assess the impact of a clinical pharmacy medication review to improve patient safety in frail older adults at risk of polypharmacy.	Medication optimisation, deprescribing and harm prevention 1,300 patients were reviewed with 24% (*n* = 306) identified as moderately to severely frail. Initiation of medication to address previously unmet health needs occurred in 12% of the reviews. 11% of the assessments led to referrals to other primary care services, and 9% of patients had high-risk medicines deprescribed. 4.5% of reviewed patients were high risk of readmission. Authors stated medication reviews of patients at risk of hospital admission had several benefits. Home visits improved access for patients to specialist medicines advice and reduced polypharmacy.	Enhancing models of care – medication reviews for frail patients.

*PIPS *Pharmacist independent prescribers, *RCT* randomised controlled trial, *CHIPPS* The Care Home Independent Pharmacist Prescriber Study, *COPD* Chronic obstructive pulmonary disease, *LTCs* long term conditions, *AF* atrial fibrillation, *HCPs* health care professionals, *CCG* Clinal Commissioning Group, *MOCH *Medicines Optimisation in Care Homes, *GPs* General Practitioners

A summary of the clinical roles delivered by pharmacists is noted in Table 3.

### The role of clinical pharmacists and services delivered for older people and people with dementia

The literature suggested the foremost activity of clinical pharmacists in supporting older people is completing medication reviews and addressing risks of polypharmacy [29, 30, 32, 34–37, 39–44, 48–52, 54, 58, 59]. They optimised processes such as reconciliation, ordering, storage, prescribing, deprescribing, monitoring, and medication administration [29, 30, 32, 33, 37, 39–45, 48, 50, 56, 58, 59]. Clinical pharmacists are also tasked with developing pharmaceutical care plans (PCPs) which identify individual needs and risks and have clearly defined therapeutic goals [32, 35, 47, 51].

At the organisational level, clinical pharmacists participated in multi-disciplinary team meetings [49, 50, 57] and supported training for staff and patient education [32, 38, 50, 52, 53]. They may conduct home visits, provide services within GP practices and care homes [33, 35, 39, 56, 58], and offer emergency out-of-hours care [43, 57].

Increased clinical pharmacist involvement improved patient safety and care through evidence-based prescribing [31, 36, 39, 44, 51]. They educated staff and care home managers on medications and optimised administration for enhanced patient safety [30, 31, 39, 40, 56]. Clinical pharmacists led audits, identifying frail, high-risk patients for disease management and reduced polypharmacy risk [30, 35, 37, 39, 51, 53, 55, 56, 58]. The aim for many pharmacist-led interventions is to enhance overall well-being and safety by ensuring that patients are receiving the appropriate medications for their specific health conditions [32, 35, 37–39, 43, 47, 50, 52, 55, 58]. Clinical pharmacists also contributed to NHS cost effectiveness through assisting with reductions of hospitalisations and contributed to financial savings from deprescribing medications [30, 32–34, 36, 48, 49, 52, 54, 57].

The literature offered limited insights into how clinical pharmacists specifically support people with dementia. While research indicates that care home-based clinical pharmacists provided support to residents with cognitive impairment [31, 53], their services appeared to be similar to those offered to older people without dementia, particularly deprescribing and medication optimisation duties [29, 30, 32, 34–36, 39–44, 48–52, 54, 58, 59].

**Table 3:** Identified clinical pharmacist roles and key tasks in the service they deliver

Role	Services delivered
Medication management	**Creation of pharmaceutical care plans (PCPs)** [32, 35, 47, 51] **Medication reviews for optimisation and full medication management services** [29, 30, 32, 34–37, 39–44, 48–52, 54, 58] **Medication reconciliation** [32, 37, 42] **Organising repeat prescriptions** [44] **Prescribing and authorisation of prescriptions** [31–33, 42, 51, 52, 56] **Reviewing polypharmacy** [34, 36, 40, 48, 52–56, 58, 59] **Deprescribing** [29–31, 42, 43, 45, 48, 50, 51, 53, 58, 59]
Education	**Providing patient education** [52, 53] **Providing support to patients with long-term conditions** [33, 35–37, 42] **Assisting in training of clinical staff and care home managers/staff** [32, 56] **Signposting of patients to other services** [53]
Organisational	**Contributing to multi-disciplinary teams** [49, 50, 57] **Working in out of hours emergency services** [57] **Making referrals to other health care professionals** [33, 51, 58] **Undertaking home visits** [33, 35, 39, 56, 58]

### Perceptions of stakeholders

Several articles discussed stakeholders’ views on clinical pharmacists supporting older people, emphasising their impact on medication management, LTC oversight, and other staff’s workload. Stakeholders include GPs, care home staff, NHS nurses, and pharmacists [30, 31, 35, 40, 42, 44, 45, 47–56].

#### Medication and long-term condition management

Stakeholders expressed enthusiasm for clinical pharmacists conducting medication reviews [35, 40, 42, 48, 49, 52, 54]. GPs recognised clinical pharmacists as valuable and previously underutilised sources of information on medication interactions and pharmaceutical guidelines [42, 44, 49], however despite this positive outlook, barriers existed. Patients and stakeholders still perceived GPs as having overall responsibility for medication, especially if clinical pharmacists were not part of the GP practice [31, 42]. When not employed by the GP practice, clinical pharmacists showed reduced willingness to take on certain responsibilities, such as ordering repeat prescriptions [42]. Concerns were also raised by GPs about potential detachment from patients if clinical pharmacists assumed a larger role in medication management [51].

Some GPs expressed doubts about clinical pharmacists managing medication, citing concerns about their knowledge and clinical competence in prescribing and deprescribing for older people [31, 44]. Authors also noted variations in the level of detail during reviews with patients when completing PCPs, as reported by GPs [31, 51, 55]. While there was recognition of clinical pharmacists’ specialist knowledge in medication interactions and guidelines [40, 44], GPs questioned their experience and understanding of care homes and the clinical needs of older people [31, 44, 50].

Transparency and a clearly defined role for clinical pharmacists, along with mutual understanding of responsibilities and boundaries, were deemed vital to carry out efficient clinical duties [31, 44]. This reduced the risk of duplicate orders and omissions in medication responsibilities among healthcare professionals supporting the same patients [44]. Stakeholders emphasised the importance of autonomy and independence for clinical pharmacists, coupled with cooperative skills, to enhance patient services [44, 49, 51].

#### Staff workload

Reports indicated that clinical pharmacists effectively reduced GP workload and staff time, a positive outcome acknowledged by stakeholders [31, 46, 51, 54]. GPs were particularly supportive of clinical pharmacists taking on responsibilities such as medication reviews and repeat prescription orders, as it allowed them more time for patient appointments and other patient-facing duties [44, 49, 51, 54], addressing the substantial time GPs typically spend resolving medication issues [56].

In care homes, clinical pharmacists managed medication tasks and deprescribing, resulting in a reduced workload for nursing staff with fewer medications to administer [30]. Care home managers appreciated the time-saving impact, as clinical pharmacists reduced the need for dose approval and verification [51]. The on-site presence of clinical pharmacists facilitated prompt responses to queries, benefiting both staff and GPs [31, 44].

Some concerns were raised by GPs regarding clinical pharmacists initiating or deprescribing medications and potential implications for their workload, including the perceived need for authorisation [42]. Reassurance was sought to ensure that their workload would not increase [44]. Suggestions were made to leverage IT systems to support deprescribing tasks, with one study proposing that a clinical pharmacist intervention using digital technology could be easily adopted [38]. Barriers to implementation were noted in the absence of robust IT systems and inaccessible electronic GP records [31, 44, 49, 52].

#### Patient safety and knowledge of older people

Skills, knowledge, and confidence were required for clinical pharmacists to respond to issues in reviews of high-risk patients, providing expert advice and support [35, 40, 44, 55]. Professional competence in deprescribing, emphasising team decision-making, was considered vital [50]. Specific knowledge of older people and mandatory guidelines in care homes were also deemed fundamental [43–45, 47]. Input from geriatricians and discussions on case studies improved clinical pharmacists’ understanding of ‘geriatric medication’ [31].

#### Team dynamics

At the interpersonal level, clinical pharmacists promoted collaboration between care homes, GP practices, community pharmacists, patients, and residents, leading to reports of strengthening the primary care team and facilitating multidisciplinary care coordination [33, 40, 44, 49, 51]. The role of the clinical pharmacist was welcomed on the basis that equal collaboration across stakeholders was demonstrated [44]. They were seen as beneficial in overcoming communication gaps between stakeholders particularly when managing patients with LTCs and complex medication-related needs, such as interactions and side effects [35, 44]. Recognition of the need to establish effective relationships was an important element for clinical pharmacists’ acceptance within a team and successful collaboration among care home staff, GPs, and community pharmacists [44, 47, 49, 56]. Consideration was suggested for training that equips clinical pharmacists with higher-level management skills, to facilitate trusting relationships within multidisciplinary teams [56].

Building and maintaining stakeholder-clinical pharmacist relationships required trust and clear communication [40, 42–44, 49, 51]. Clinical pharmacists needed to understand their colleagues, local cultures, communication preferences, and boundaries to facilitate collaboration [47]. Those embedded in the GP practice or care home encountered fewer communication barriers and engaged in more deprescribing activities than clinical pharmacists who were not embedded [29, 31]. Established working practices were beneficial for effective communication, emphasising the importance of workplace relationships for improved patient outcomes. Strong communication skills were prioritised, particularly when supporting care home residents with cognitive impairment [44].

### Perceptions of older people and people with dementia

Older people appreciated the personal approach, guidance, and detailed explanations from clinical pharmacists [33, 38, 51, 53] and it was valued when care home residents and/or families were fully involved in reviews and medication decisions [49]. Patients felt more confident and informed about medications, valuing the opportunity for questions and in-depth discussions [52, 53]. Some preferred the general practice setting with a clinical pharmacist over community pharmacies due to concerns about privacy and commercialisation [46].

However, confusion among patients persisted regarding clinical pharmacists’ services and if they could prescribe, suggesting a preference for medication changes to remain with GPs [31, 42]. Care home managers proposed staff training to clarify the clinical pharmacist service for better acceptance by residents and their relatives [44].

The availability of sufficient time to engage with patients during medication reviews is highly valued [31]. Time constraints posed a significant barrier especially for undertaking clinical duties like prescribing [42, 50] and can result in poor communication and overlooking individual patient needs [43]. Sufficient time in appointments enabled clinical pharmacists to address concerns, questions, and misconceptions [31, 42] which were crucial to facilitate patient engagement, acceptance of changes, and addressing patient needs [43]. Moreover, clear communication about medication changes was crucial, as some patients felt their medication was stopped as a cost-saving measure rather than for their benefit [42].

Articles reported positive feedback from patients who had the ability to ask questions during a review [31, 50] and having sufficient time in appointments developed relationships between the patient and clinical pharmacist [42], particularly when reviews were conducted in-person [51]. It was suggested that a 20–30-minute appointment provides opportunity to develop a comprehensive understanding of a patient’s needs and support deprescribing [55].

While positive feedback was received from care home residents with communication difficulties, patients with impaired cognition viewed the clinical pharmacy service as less useful [31, 53]. Additionally, non-English speakers highlighted communication challenges [53]. The exploration of these aspects faced by those from minority ethnic backgrounds lacked depth in the articles however, with only one briefly addressing these challenges at hand [53].

## Discussion

### Summary

This scoping review maps the various roles, activities, and settings of clinical pharmacists providing support for older people and people with dementia in primary care. The findings enhance our understanding of clinical pharmacist roles and services in the UK, as well as highlight the gaps in our understanding. This can inform policy development and future research within the context of NHS provision and commissioning for older people and people with dementia.

The review suggests that clinical pharmacists, particularly when embedded in GP practices or care homes, hold promise in supporting the growing ageing population. Their services have the potential to enhance primary care collaboration, increase knowledge among staff and patients, improve patient safety, reduce staff workload, and cut NHS expenditures due to their prominent role in deprescribing. For a collegiate service, clinical pharmacists require clearly defined roles understood by colleagues and patients, facilitated by clear and consistent communication, and the development of trusting relationships among stakeholders. While there is insufficient evidence on the impact of clinical pharmacists in supporting older people with cognitive impairment, there is reason to believe they could also benefit from such services.

### Strengths and limitations

There are strengths in our review, which include the comprehensive database searches for peer-reviewed and grey literature, along with a broad inclusion criterion. Limitations include potential incorporation of low-quality, biased evidence, due to the lack of quality assessments, and the lack of full double screening of all papers, as only 10% of papers underwent this process. However, these approaches follow guidance for conducting scoping reviews [59]. Moreover, the concept polypharmacy/medication management were utilised in our search strategy to enhance specificity to services provided by clinical pharmacists, but this may have excluded potential services outside of medication management.

### Implications for policy and practice in the UK

Clarity and communication about clinical pharmacists’ roles and training may be able to reassure patients and other stakeholders. Studies in health communication have established an association between clinical-patient communication and improved patient outcomes, such as mutual understanding and adherence [60]. Hence, healthcare systems should enhance awareness among stakeholders, patients, and the public that prescribing and deprescribing are professional roles carried out by clinical pharmacists. To address varying levels of detail in PCPs, enhanced training with standardised templates to ensure consistency may be needed. Given that knowledge of older people’s medicines and conditions is crucial for a successful clinical pharmacist service, incorporating training in clinical care of older people, including dementia, is recommended. This is particularly important as concerns were raised about clinical pharmacists’ understanding of older people and medications in care homes. Limited knowledge of dementia is reported as a barrier to optimal care by health professionals [61], and thus formal education and professional development is needed to better prepare clinical pharmacists to provide quality care to older people and people with dementia. Moreover, when carrying out medication management duties, clinical pharmacists’ access to electronic patient records in real time appears to be crucial. This would help ensure they are equipped with the necessary tools to carry out their clinical duties effectively.

### Implications for future research

Future research should explore the views and experiences of people with dementia and their family members or carers to shape policies that support inclusive clinical pharmacist services for all older adult populations. This research can identify challenges and propose policy solutions to facilitate the expansion of clinical pharmacist-led services. Additionally, empirical research among minoritised ethnic groups is necessary to address cultural and linguistic influences, as evidence has demonstrated patients from ethnic minority groups are less likely to engage in medicine reviews and report poorer adherence to medication than their white counterparts [62]. Our research identified significant gaps in this area of research. Thus, further efforts are needed to understand the barriers faced by individuals with communication impairments or those where English is not their first language, to facilitate effective relationships between clinical pharmacists, and patients and carers. Investigating the policy implications of integrating clinical pharmacists into community care for all populations is vital, along with understanding barriers to receiving clinical pharmacist support.

Further research is needed to quantify the impact of pharmacist-led interventions on outcomes such as medication adherence, hospitalisations, cost-effectiveness, and health-related quality of life for older people and their carers. Clarification on the cost-effectiveness and health economics related to deprescribing, reduced hospitalisations, and the burden on secondary care services is necessary. Cost-benefit analyses can provide insights to inform NHS healthcare policy decisions on resource allocation.

## Conclusions

There is evidence that clinical pharmacists play an important role in primary care teams that extends beyond direct patient care and influences the broader healthcare landscape. We have outlined key components for the implementation of an effective service to take place, which provides valuable insights for policy development within the context of NHS provision and may be transferable to international policy and practice.

## Supplementary Information

Supplementary Material 1.

## Data Availability

All data generated or analysed during this study are included in this published article [and its supplementary information files].
