# Supplementary material for: Service delivery and the role of clinical pharmacists in UK primary care for older people, including people with dementia: a scoping review
Source: BMC Prim Care. 2025 Jan 14;26:10. doi: 10.1186/s12875-024-02685-x (PMC11731431; doi:10.1186/s12875-024-02685-x)
Supplement: Supplementary file 1 — Supplementary Material 1. [file 12875_2024_2685_MOESM1_ESM.docx]

Appendix (1)

Full electronic search strategy for Medline, Web of Science and CINAHL.

| Medline search | |
| --- | --- |
| Search | Term |
| 1 | exp "Aged, 80 and over"/ or exp Frail Elderly/ or (aged or elderly).mp. or (Elderly or "Health Services for the Elderly").mp. or (Old age or Elderly population group or Frail Elderly).mp. |
| 2 | exp General Practice/ or exp Family Practice/ or (general practice field or General Practitioners).mp. |
| 3 | exp Primary Health Care/ or (care, primary or care, primary health or health care, primary or healthcare, primary or primary care or primary health care or primary healthcare).mp. or (Primary Health Care or Primary Care Physicians or Primary care provider or Primary care consultation or primary care facility).mp. |
| 4 | exp Pharmacists/ or (clinical pharmacist or clinical pharmacists or pharmacist or pharmacist, clinical or pharmacists or pharmacists, clinical).mp. |
| 5 | exp Medication Therapy Management/ or exp Medication Errors/ or exp Drug Prescriptions/ or (drug therapy management or management, drug therapy or management, medication therapy or medication therapy management or therapy management, drug or therapy management, medication).mp. or (drug prescribing or drug prescribings or drug prescription or drug prescriptions or prescribing, drug or prescribings, drug).mp. |
| 6 | exp Polypharmacy/ or exp Drug Therapy, Combination/ or exp Pharmaceutical Preparations/ or (polymedication or polypharmacy).mp. or (drug or drugs or pharmaceutic preparations or pharmaceutical or pharmaceutical preparation or pharmaceutical preparations or pharmaceutical product or pharmaceutical products or pharmaceuticals or preparation, pharmaceutical or preparations, pharmaceutic or preparations, pharmaceutical or product, pharmaceutical or products, pharmaceutical).mp. or (Polypharmacy or Polypharmacy).mp. or (Polypharmacy or "Assessment of Patient Status Related Polypharmacy" or "At risk of polypharmacy").mp. |
| 7 | 2 or 3 |
| 8 | 5 or 6 |
| 9 | 1 and 4 and 7 and 8 |

| Web of Science search |
| --- |
| **((((ALL=(elderly or aged or older or elder or geriatric or elderly people or old people or old people or senior)) AND ALL=(general practice or gp or primary care or primary healthcare or primary health care)) AND ALL=(medication management or medication adherence or medication compliance)) OR ALL=(polypharmacy or multiple drugs or medications)) AND ALL=(clinical pharmacist)** and **ENGLAND** or **WALES** or **SCOTLAND** or **IRELAND** or **NORTH IRELAND** (Countries/Regions) |

| CINAHL search |
| --- |
| (elderly or aged or older or elder or geriatric or elderly people or old people or old people or senior ) AND ( general practice or gp or primary care or primary healthcare or primary health care ) AND ( medication management or medication adherence or medication compliance ) OR ( polypharmacy or multiple drugs or medications ) AND clinical pharmacist" |

Appendix (2)

| Example earch terms and categories used for Medline. These terms were adapted for use across all databases. | |
| --- | --- |
| **Category** | **Search terms** |
| Primary care | primary care or primary health care or primary healthcare or primary care provider* or primary care facilit* or general practice or general practitioner or exp Primary Health Care/ or exp General Practice/ |
| Clinical pharmacist | clinical pharmacist* or pharmacist* or exp Pharmacists/ |
| Older people | age Factors/ or age 65 and over or age* or elderly or frailty or older adult* |
| Medication management | medication management or drug therap* or medication or polypharmacy or exp Medication Therapy/ or exp Polypharmacy/ |
